# Remedies and Their Uses

**Published:** 1907-06-29

**Authors:** 


### Remedies and their Uses.

### Syrup Trifolium Co.

This is a combination of iodide of potassium with various vegetable extracts, the pharmacological action of which is summed up in the vague term "alterative." It is, however, a useful preparation in syphilitic and other conditions where iodides are usually given. It is palatable and easily taken by children. For this reason it may be combined with liquor hyd. perchlor. in the treatment of congenital cases. The amount of iodide present is not large, and additional amounts may be added if necessary. Calcium sulphide grains 16, potassium iodide ʒj., syrupus trifolium co. ad ʒviij. is recommended as a useful mixture in tertiary syphilis with skin lesions. Drachm doses may be ordered thrice daily, and increased to 3 drachms. The doses should, of course, be well diluted. The syrup is supplied by Parke Davis and Co.

*Mergal* is a mixture of oxycholate of mercury and tannate of albumin, and is sold in capsules containing $\frac{3}{4}$ grain of the mercury salt. Leistikow recommends it in cases which are intolerant of ordinary mercurials, as the substance is not a gastro-intestinal irritant. He has used it in 20 cases of primary and secondary syphilis, and considers it may still be given in small doses when some stomatitis is present. It is manufactured by J. D. Riedel, Berlin.

*Ecthol* is intended for external as well as for internal use, and is a compound containing extracts of chinacea and thuja. It is said to be of value as an application in tertiary syphilis, and appears to be principally employed when pus formation or gangrene has occurred. Thus it has been applied to pustular eruptions such as small-pox, severe chicken-pox, etc. When given internally the dose is one teaspoonful, three times daily; as an external application the fluid should be well rubbed in as often as necessary. It is prepared by Battle and Co.

